# Primary Cilia Blockage Promotes the Malignant Behaviors of Hepatocellular Carcinoma via Induction of Autophagy

**DOI:** 10.1155/2019/5202750

**Published:** 2019-10-02

**Authors:** Lian Liu, Jia-Qi Sheng, Mu-Ru Wang, Yun Gan, Xiao-Li Wu, Jia-Zhi Liao, De-An Tian, Xing-Xing He, Pei-Yuan Li

**Affiliations:** Division of Gastroenterology, Tongji Hospital, Tongji Medical College, Huazhong University of Science and Technology, Wuhan, China

## Abstract

Primary cilia are organelles protruding from cell surface into environment that function in regulating cell cycle and modulating cilia-related signal. Primary ciliogenesis and autophagy play important roles in tumorigenesis. However, the functions and interactions between primary cilia and autophagy in hepatocellular carcinoma (HCC) have not been reported yet. Here, we aimed to investigate the relationship and function of primary cilia and autophagy in HCC. *In vitro*, we showed that serum starvation stimuli could trigger primary ciliogenesis in HCC cells. Blockage of primary ciliogenesis by IFT88 silencing enhanced the proliferation, migration, and invasion ability of HCC cells. In addition, inhibition of primary cilia could positively regulate autophagy. However, the proliferation, migration, and invasion ability which were promoted by IFT88 silencing could be partly reversed by inhibition of autophagy. *In vivo*, interference of primary cilia led to acceleration of tumor growth and increase of autophagic flux in xenograft HCC mouse models. Moreover, IFT88 high expression or ATG7 low expression in HCC tissues was correlated with longer survival time indicated by the Cancer Genome Atlas (TCGA) analysis. In conclusion, our study demonstrated that blockage of primary ciliogenesis by IFT88 silencing had protumor effects through induction of autophagy in HCC. These findings define a newly recognized role of primary cilia and autophagy in HCC.

## 1. Introduction

Hepatocellular carcinoma (HCC) is one of the most common malignant tumors, which ranks second in cancer deaths worldwide [1]. Up to now, surgical therapy remains the first choice for the treatment of HCC. The prognosis of HCC patients is still poor due to the high recurrence rate and distant metastasis [2]. It is still imperative to explore the pathogenesis and therapeutic targets of HCC at present.

The primary cilia are microtubule-based organelles that function in regulating cell cycle and modulating signal transduction [3–5]. Since the basal body of primary cilium derives from the centrosome or mature centriole, it must be disassembled before mitosis to release the tackled centriole and then to contribute to the formation of spindle [4, 6]. In this way, the presence of cilium can suppress abnormal cell growth by delaying cell-cycle progression to the S or M phase [7, 8]. The intraflagellar transport (IFT) process is essential for the assembly and maintenance of cilium, as it anterogradely or retrogradely delivers building blocks to the ciliary assembly site at the tip of the cilium. IFT proteins function in different aspects of the IFT process and have been dissociated into two smaller subcomplexes (IFT-A and primary IFT-B) [9]. IFT88 is one of the core proteins of IFT-B complex, the mutation of which can lead to absent or shortened cilia [10, 11]. Primary cilia function as signaling hubs of multiple signaling pathways relevant to cancer including Sonic Hedgehog (Shh) signaling [12–14], Wnt (canonical and noncanonical) signaling [15, 16], platelet-derived growth factor (PDGF) signaling [17], or fibroblast growth factor (FGF) pathways [18–21]. Since primary cilia have the ability to delay cell cycle progression and to modulate signal transduction, the connection between primary cilia and tumorigenesis has been proposed as a new clue for cancer research [7, 22]. Clinical data showed that primary ciliogenesis was weakened in multiple human cancers including breast cancer, renal cell carcinoma, melanoma, and pancreatic cancer [23–26]. Although defective cilia and cancer are always associated, the mechanism of cilia on tumorigenesis is still unclear. As far as we know, the relationship between primary cilia and HCC has not been reported yet.

Autophagy is an evolutionarily conserved lysosome-dependent catabolic process which degrades cell's components in order to recycle substrates to adapt to tough circumstances [27]. It is a critical cellular homeostatic mechanism with stress resistance, immunity, antiaging, and protumor or antitumor effects [27]. Basal autophagy acts as a tumor suppressor by maintaining genomic stability in normal cells. However, once a tumor is established, unbalanced autophagy will contribute to carcinoma cell survival under tumor microenvironment and in turn promote tumor growth and development [28–30]. The dynamic role of autophagy can also apply on HCC [31, 32].

Is there any correlation between primary cilia and autophagy? Nutrient deprivation is a stimulus shared by both primary cilia and autophagy. Recent research found that basal autophagy negatively modulated primary ciliogenesis by removing essential ciliogenesis proteins, while activated autophagy promoted ciliary growth by degradation of oral-facial-digital syndrome 1 (OFD1, a negative ciliogenesis regulatory protein) in MCF7 breast cancer cells [33–35]. Interestingly, in mouse embryonic fibroblasts (MEFs) or kidney epithelial cells (KECs), primary cilia may be necessary for starvation-induced autophagy reversely [33]. Although recent studies have reviewed the dual interplay between autophagy and primary cilium [36–38], research studies about their relationship in cancer cells remain scarce. Likewise, whether primary cilia and autophagy involve in hepatocellular carcinogenesis has not been reported before.

In the present study, we found that the primary ciliogenesis could be induced by serum starvation, and the blockage of primary ciliogenesis by IFT88 silencing promoted the malignant behaviors of HCC via induction of autophagy.

## 2. Materials and Methods

### 2.1. Cell Culture

The HCC cell lines SMMC-7721, HCC-LM3, Huh7, MHCC97-H, SK-Hep-1, HepG2, and Hep3B were stored in the Institute of Liver Diseases (Tongji Hospital, Wuhan, China) and were resuscitated and cultured in DMEM containing 10% fetal bovine serum (FBS) (Invitrogen, USA) or serum-free medium in a 5% CO_2_ incubator at 37°C.

### 2.2. Cell Transfection and Infection

Transfection of siRNA was performed using the Lipofectamine 3000 reagent (Invitrogen, USA) according to the manufacturer's instruction. siRNA targeting human IFT88/ATG7 was synthesized and purified by RiboBio (Guangzhou, China). The sequence of siRNA oligonucleotides is shown in Supplementary [Supplementary-material supplementary-material-1]. A scrambled siRNA was used as negative control. Cells were transfected with 20 nmol/L siRNA after seeding and were changed to fresh medium with 10% FBS 6 hours later. The RFP-GFP-LC3 double fluorescence lentivirus was purchased from Genechem (Shanghai, China). Cells cultured in 6-well plates were infected with 3.0 × 10^8^ units/ml lentivirus at a MOI (multiplicity of infection) of 10 and then selected with puromycin (1 *μ*g/ml) according to the manufacturer's instructions.

### 2.3. RNA Extraction and Real-Time PCR

Total RNA was extracted using TRIzol reagent (Invitrogen, USA) according to the manufacturer's instructions. Reverse-transcribed complementary DNA was synthesized using the Prime Script RT Reagent Kit (Takara, Japan). Real-time PCR was performed as we have described before [39]. The sequences of the primers used for RT-qPCR are listed in Supplementary [Supplementary-material supplementary-material-1]. Difference between samples was determined by the 2^−ΔΔCt^ method.

### 2.4. Western Blot

Western blot was performed as we have described before [40]. The primary antibodies used were anti-*β*-actin and anti-IFT88 (Proteintech, China) and anti-p62, anti-ATG7, and anti-LC3A/B (Cell Signaling, USA). The secondary antibody used was anti-rabbit IgG (Jackson ImmunoResearch Laboratories, USA). Blot was scanned, and densitometric analysis was done by ImageJ software (NIH, USA).

### 2.5. Immunofluorescence Staining

Cells were seeded and cultured on coverslips. After treatment for the indicated time, cells were fixed in 4% paraformaldehyde for 15 min and incubated in blocking solution (10% donkey serum and 0.1% Triton X-100 in PBS) for 1-2 hours at room temperature. Cells were then incubated with primary antibody (mouse anti-acetylated *α*-tubulin; Sigma, USA) overnight at 4°C in the cold room followed by the secondary antibody (Alexa Fluor 488 AffiniPure Donkey Anti-Mouse IgG; Jackson ImmunoResearch Laboratories, USA) for 2 hours at room temperature under dark conditions. Finally, cells were stained with DAPI (4′,6-diamidino-2-phenylindole) and mounted with antifluorescence quencher (Promotor, China). Fluorescence images were directly taken using an inverted confocal microscope (Olympus, Japan).

### 2.6. Cell Proliferation, Migration, and Invasion Assay

Cell proliferation was determined using the Cell Counting Kit-8 (CCK-8, Promotor, China) according to the manufacturer's instructions. CCK-8 reagent was added in the 96-well plates at 37°C for 2 hours to detect the optical density (OD) value at 450 nm. The migration and invasion assays were performed as described before by using transwell insert chambers (8 mm pore size, Corning, USA) [40].

### 2.7. Transmission Electron Microscope (TEM)

Cells were fixed with 2.5% glutaraldehyde in cold PBS. The specimens were postfixed in 1% osmium tetroxide with 0.1% potassium ferricyanide, dehydrated through a graded series of ethanol (30%–90%), and embedded in Epon. Ultrathin sections (65 nm) were stained with 2% uranyl acetate and Reynold's lead citrate. Images were taken on a HITACHI HT7700 TEM system (HITACHI, Japan) at 80 kV.

### 2.8. RFP-GFP-LC3 Autophagic Flux Assay

Cells infected with RFP-GFP-LC3 double fluorescence lentivirus were cultured on cover slips in 24-well plates. Fluorescence images were directly taken using an inverted confocal microscope (Olympus, Japan). Autophagic flux was examined by analyzing the formation of fluorescent puncta of autophagosomes and autolysosomes in cells.

### 2.9. Animal Experiments

All animal experiments were performed in accordance with protocols approved by the Ethics Committee of Tongji Hospital. Male BALB/c nude mice (12–14 g, 3-4 weeks old) were obtained from Beijing Vital River Laboratory Animal Technology. Mice were bred in pathogen-free conditions at 22 ± 2°C with 60% ± 5% relative humidity under a 12 h light and dark cycle with free access to water and regular chow diet. For xenograft models, 5 × 10^6^ SMMC-7721 cells in 0.1 ml serum-free DMEM were injected into the subcutaneous region of nude mice. After 10 days, the transplanted nude mice were randomly divided into two groups (*n* = 5 each). Cholesterol-modified si-NC or si-IFT88 was directly injected into the implanted tumor at the dose of 10 nmol every 3 days for five times. Tumor volume (V) was calculated with the formula V = (L × W^2^) × 0.5.

### 2.10. Histology

All mice were euthanized by pentobarbital natrium and sacrificed by a cervical vertebrae luxation, and then the tumors were taken and embedded in paraffin. The slides were sectioned and stained with H&E. Tumor tissues were also used for IFT88 and Ki-67 immunohistochemical staining. The procedures were performed as previously described [40].

### 2.11. Survival Analysis

We used the Cancer Genome Atlas (TCGA) database to perform Kaplan–Meier survival analysis as we have described before [41]. The low expression was defined by expression levels in the lowest quartile (25th percentile) and high expression by levels in the upper quartile (75th percentile). The numbers of high-expression and low-expression samples of IFT88 are both 93 while those of ATG7 are 50. *P* value was calculated by log-rank (Mantel–Cox) test. *P* < 0.05 was considered significant statistical difference.

### 2.12. Statistical Analysis

All experiments were performed in triplicate unless specified. Results were represented as the mean ± SEM. Statistical analysis was performed using unpaired Student's *t* test. *P* < 0.05 was considered significant.

## 3. Results

### 3.1. Serum Starvation Induces Primary Ciliogenesis in HCC Cells

**Figure 1:**
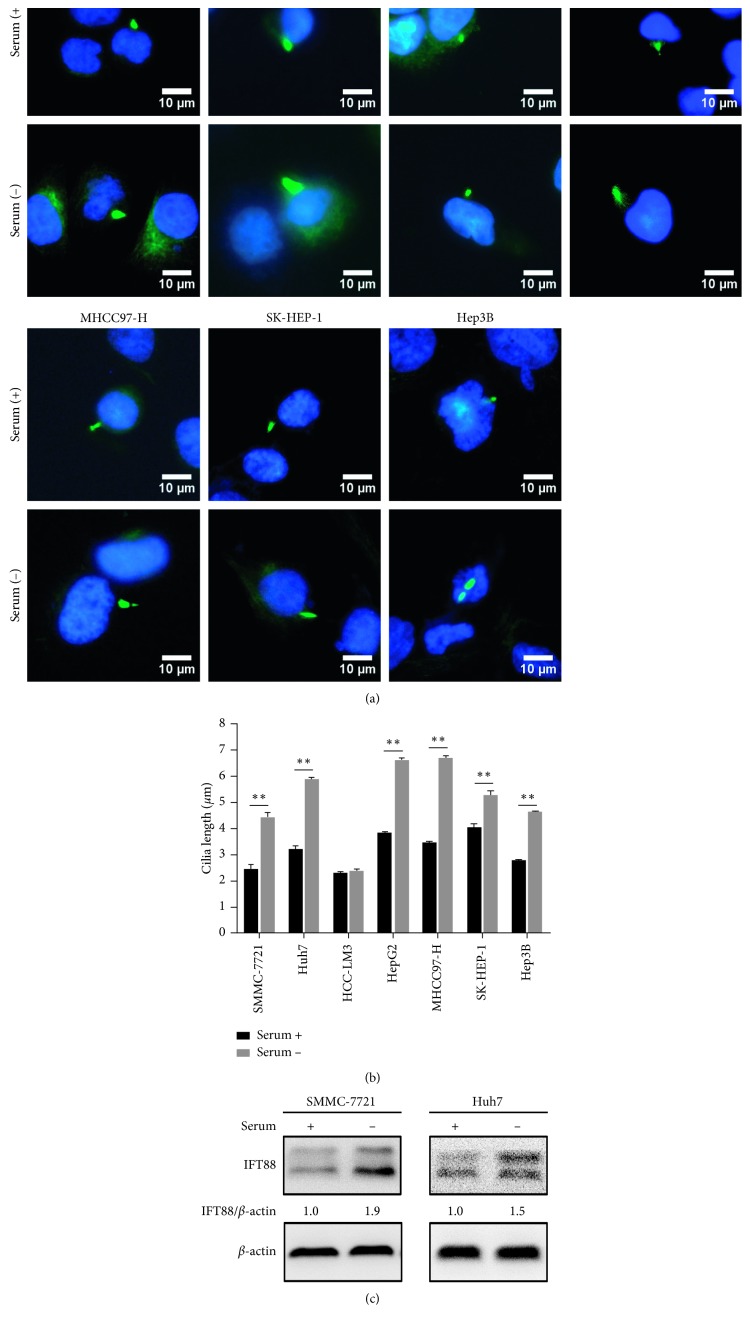
Serum starvation induces primary ciliogenesis in HCC cells. (a) Representative immunofluorescence images of cilia marker acetylated tubulin in SMMC-7721, Huh7, HCC-LM3, HepG2, MHCC97-H, SK-Hep-1, and Hep3B HCC cell lines cultured in medium with or without serum for 24 h (×200). (b) Length of primary cilia in seven HCC cell lines after serum starvation for 24 h. (c) Expression level of IFT88 in SMMC-7721 and Huh7 cells were determined by western blot analysis after serum starvation for 24 h; the value under the band is the ratio of the blot and normalized to control. ^*∗∗*^*P* < 0.01.

The nutrient deprivation stimulus was reported to induce primary ciliogenesis in several types of cultured cells [42]. To determine whether this phenomenon also exists in HCC cells, we cultured seven HCC cell lines SMMC-7721, Huh7, HCC-LM3, HepG2, MHCC97-H, SK-Hep-1, and Hep3B with or without serum. After 24 h of serum deprivation, immunofluorescence staining was used to observe the length of primary cilia. Compared to the control, the cilia length of the serum deprivation group increased from 27% to 48% in six HCC cell lines (mean ± SEM, *n* = 3, *P* < 0.01), while the cilia length of the HCC-LM3 cell group with serum deprivation increased slightly without statistical significance (Figures 1(a) and 1(b)). Then, we chose SMMC-7721 and Huh7 cell lines for further experiments. We detected the expression level of IFT88 by western blot. After 24 h of serum withdrawal, protein expression of IFT88 significantly increased (Figure 1(c)). These data illustrated that primary cilia could be induced under serum deprivation condition, accompanied by increased expression of IFT88 in HCC cells.

### 3.2. Primary Ciliogenesis Blockage Promotes the Malignant Behaviors *In Vitro*

**Figure 2:**
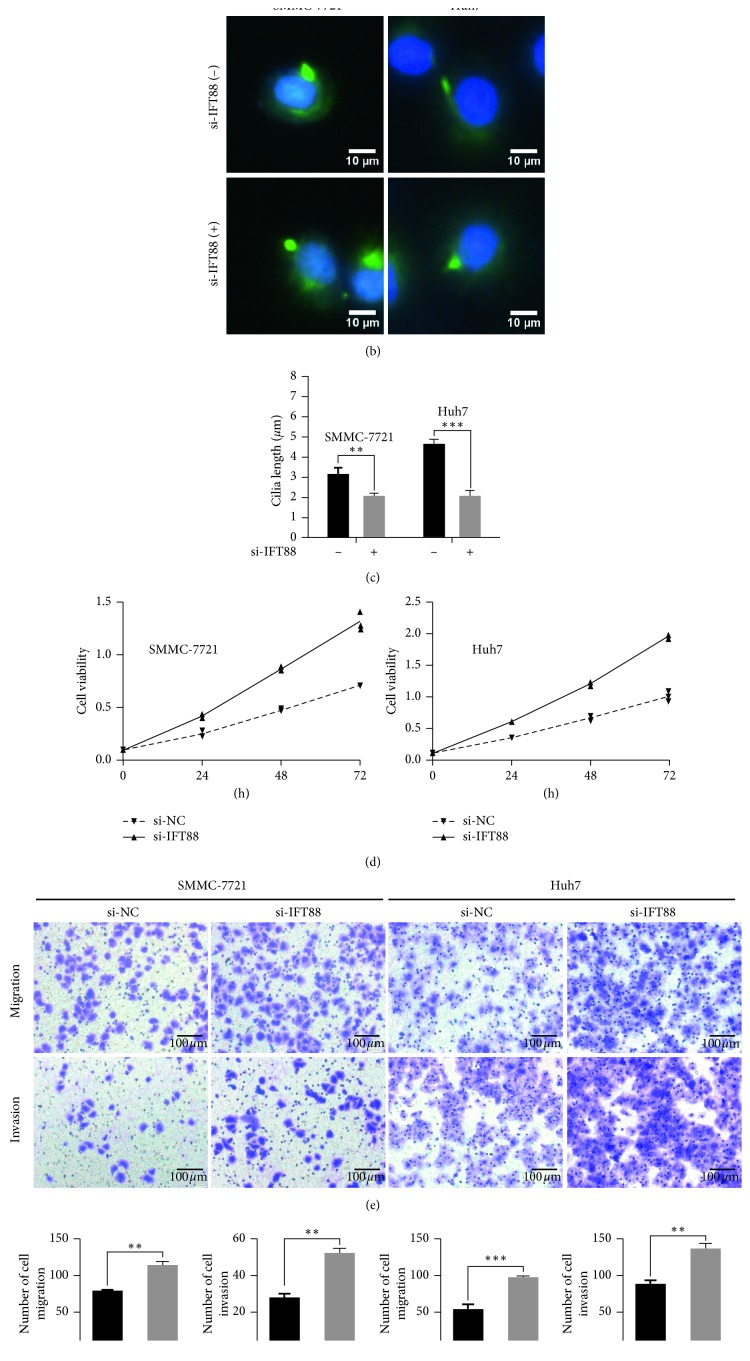
Primary ciliogenesis blockage promotes the malignant behaviors *in vitro*. (a) SMMC-7721 and Huh7 cells were transfected with si-IFT88 or si-NC (negative control) for 72 h. The expression of IFT88 was determined by western blot analysis; the value under the band is the ratio of the blot and normalized to control. (b) Representative immunofluorescence images of cilia marker acetylated tubulin (×200). (c) Length of primary cilia. (d) SMMC-7721 and Huh7 cells were transfected with si-IFT88 or si-NC (negative control) for 48 h. Cell viability was then measured by CCK-8 assay for another 24 h, 48 h, and 72 h, respectively. (e, f) Migration and invasion ability were measured by transwell assay for another 24 h (×200). ^*∗∗*^*P* < 0.01, ^*∗∗∗*^*P* < 0.001.

As described above, primary cilia have the ability to delay cell cycle progression and are often defective in multiple human cancers. So, we wanted to learn whether the blockage of primary ciliogenesis could augment the malignant behaviors of HCC cells. IFT88 is a key factor of primary ciliogenesis. Small interfering RNA (siRNA) targeting human IFT88 mRNA (si-IFT88) was selected to inhibit the mRNA and protein expression of IFT88 (Figure 2(a) and Supplementary Figures [Supplementary-material supplementary-material-1] and [Supplementary-material supplementary-material-1]). As shown in Figures 2(b) and 2(c), the cilia length decreased by 36% in SMMC-7721 cells and 55% in Huh7 cells (mean ± SEM, *n* = 3, *P*=0.0042, *P*=0.0004) after blockage of IFT88 for 72 h compared with control (si-NC). Blockage of primary ciliogenesis by si-IFT88 markedly increased the proliferation ability of SMMC-7721 and Huh7 cells (mean ± SEM, *n* = 3, *P* < 0.0001, *P*=0.0002) (Figure 2(d)). Transwell assay also indicated that the migration ability increased significantly compared with control (SMMC-7721: 113 ± 4 vs. 78 ± 2, *P*=0.0012; Huh7: 96 ± 2 vs. 53 ± 4; *n* = 3, *P*=0.0008), as well as the invasion ability (SMMC-7721: 52 ± 2 vs. 28 ± 2, *P*=0.0012; Huh7: 135 ± 5 vs. 87 ± 4; *n* = 3, *P*=0.0012) (Figures 2(e) and 2(f)). These results demonstrated that suppression of primary ciliogenesis by IFT88 silencing displayed a promalignancy effects in HCC cells.

### 3.3. Inhibition of Primary Ciliogenesis Induces Autophagic Flux

**Figure 3:**
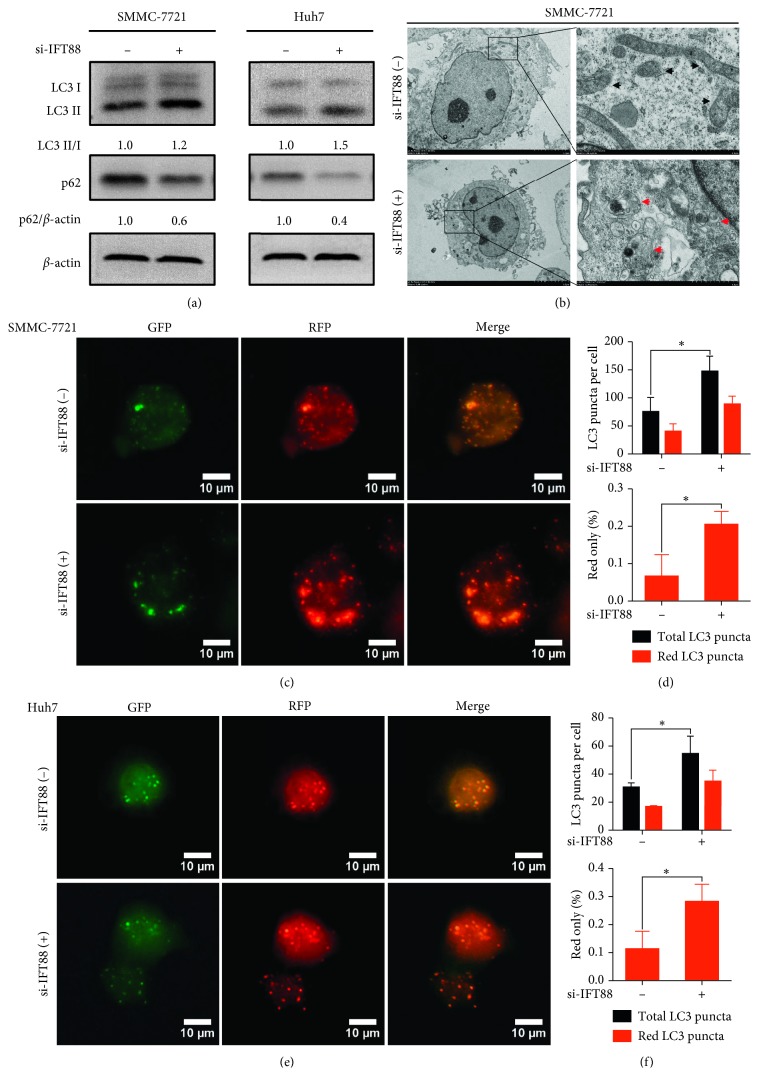
Inhibition of primary ciliogenesis induces autophagic flux. (a) SMMC-7721 and Huh7 cells were transfected with si-IFT88 or si-NC (negative control) for 72 h. The expression of LC3 II/I and p62 was determined by western blot analysis; the value under the band is the ratio of the blot and normalized to control. (b) After transfection for 72 h, SMMC-7721 cells were assayed by TEM. Autolysosomes (the rounded vacuolar structures with only one membrane containing cytoplasmic contents) are marked by red arrows. Uncleared mitochondria marked by black arrows (×1500 or ×10000). (c–f) SMMC-7721 and Huh7 cells were stably infected with RFP-GFP-LC3 double fluorescence lentivirus and then transfected with si-IFT88 or si-NC for 72 h; representative fluorescence images of GFP-LC3 puncta (green, autophagosomes) and RFP-LC3 puncta (red, autolysosomes) (×400) as well as the overlay images were shown and fluorescence puncta were counted by inverted confocal microscope. ^*∗*^*P* < 0.05.

Since inhibition of primary ciliogenesis by IFT88 silencing promotes the malignant behaviors in HCC cells, we wonder whether autophagy changes or not during this process. Interestingly, after IFT88 was silenced, the ratio of LC3 II/I increased and the expression of p62 decreased indicated by western blot (Figure 3(a)). Transmission electron microscope (TEM) assay confirmed the formation of autolysosomes after IFT88 silencing in SMMC-7721 cells, which were recognized as characteristic only one membrane vacuolar structures containing cytoplasmic contents (Figure 3(b)). Furthermore, RFP-GFP-LC3 double fluorescence lentivirus was infected into SMMC-7721 cells and Huh7 cells to observe the level of autophagic flux. This probe can be used to identify autophagosomes (GFP-positive/RFP-positive; yellow dots) and autolysosomes (GFP-negative/RFP-positive; red dots) by confocal detection because the GFP fluorescence is quantitatively quenched in low pH compartments of autolysosome [43]. As expected, the numbers of both yellow and red puncta increased after IFT88 silencing in these two HCC cell lines (Figures 3(c)–3(f)). Taken together, these results suggested that autophagic flux could be activated after blockage of primary ciliogenesis by IFT88 silencing in HCC cells.

### 3.4. Suppression of Autophagy Compromises the Promalignancy Effects of Primary Ciliogenesis Blockage *In Vitro*

**Figure 4:**
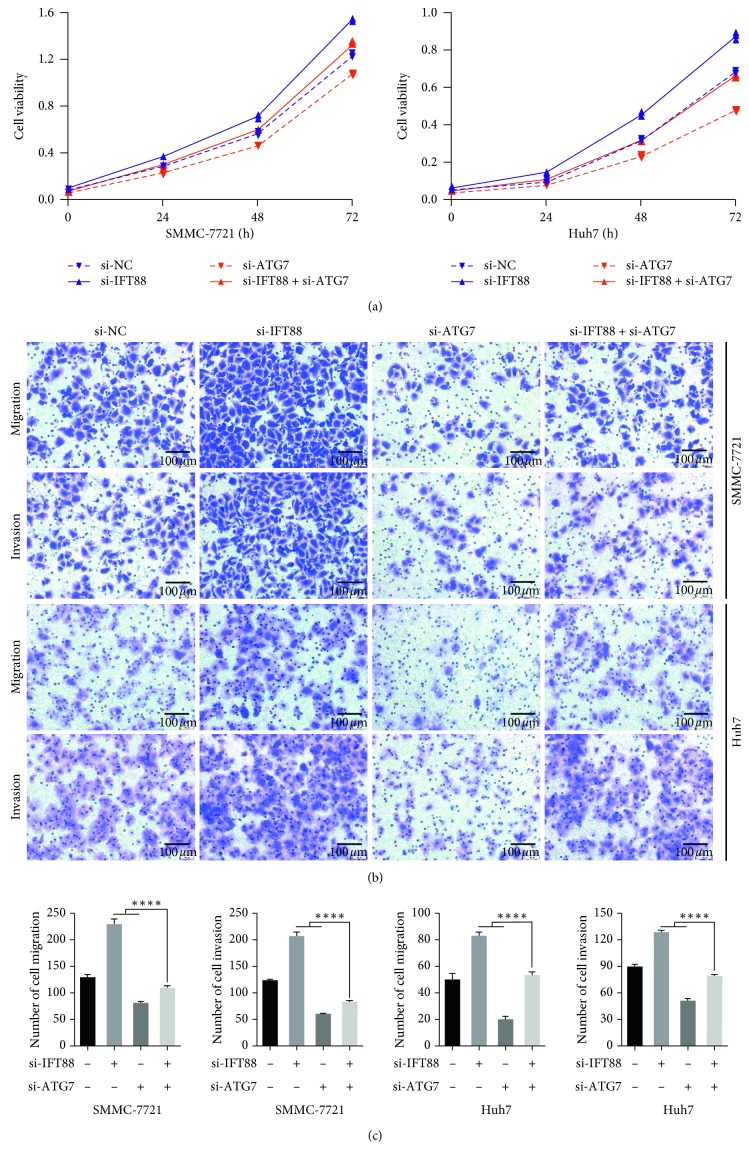
Suppression of autophagy compromises the promalignancy effects of primary ciliogenesis blockage *in vitro*. (a) SMMC-7721 and Huh7 cells were transfected with si-ATG7 and/or si-IFT88 (si-NC as negative control) to inhibit autophagy/primary ciliogenesis for 48 h. Cell viability was measured by CCK-8 assay for another 24 h, 48 h, and 72 h, respectively. (b, c) Migration and invasion ability were measured by transwell assay for another 24 h (×200). ^*∗∗∗*^*P* < 0.001, ^*∗∗∗∗*^*P* < 0.0001.

Since blockage of primary ciliogenesis by IFT88 silencing could promote the malignant behaviors and autophagic flux in HCC cells at the same time, we tried to identify the role of autophagy during the process. We selected siRNA targeting ATG7 (si-ATG7), a key autophagy gene, to suppress autophagy (Supplementary Figures [Supplementary-material supplementary-material-1] and [Supplementary-material supplementary-material-1]). si-IFT88 was used to suppress primary ciliogenesis as above. SMMC-7721 or Huh7 HCC cells were transfected with both si-IFT88 and si-ATG7 at the same time. Then, the proliferation ability was assayed by CCK8 test. Cells transfected with si-IFT88 and si-ATG7 simultaneously showed reduced proliferation ability compared with cells transfected with si-IFT88/si-NC (Figure 4(a)). Accordingly, transwell assay indicated that cells transfected with si-IFT88 and si-ATG7 showed decreased migration and invasion ability compared with cells transfected with si-IFT88/si-NC. SMMC-7721 and Huh7 showed the similar tendency (SMMC-7721: migration 228 ± 6 vs. 112 ± 1, *P* < 0.0001; invasion 205 ± 5 vs. 83 ± 1, *P* < 0.0001; Huh7: migration 83 ± 2 vs. 53 ± 2, *P*=0.0002; invasion 127 ± 1 vs. 78 ± 2; *n* = 3, *P*=0.0004) (Figures 4(b) and 4(c)). Taken together, these results suggested that inhibition of autophagy compromises the promalignancy effects of primary ciliogenesis blockage by IFT88 silencing *in vitro*.

### 3.5. Blockage of Primary Ciliogenesis Promotes Xenograft HCC Growth *In Vivo*

**Figure 5:**
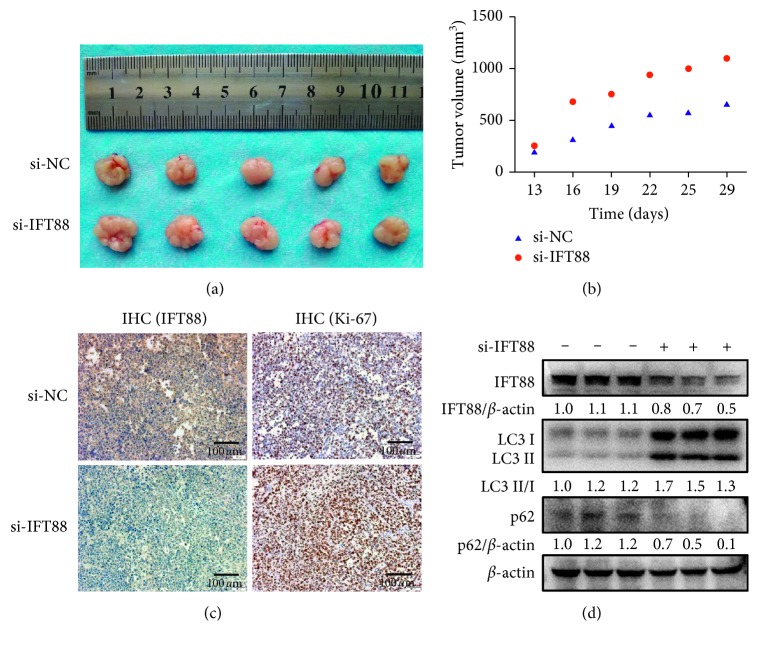
Blockage of primary ciliogenesis promotes xenograft HCC growth *in vivo*. (a) Nude mice were subcutaneously implanted with SMMC-7721 HCC cells for tumor formation and then received intratumoral injection of cholesterol-modified si-IFT88 or control (si-NC) every three days for five times. Tumors were removed three days later after the last injection. (b) Volume changes of xenograft tumors of the mouse model. (c) Immunohistochemical staining with anti-IFT88 antibody or anti-Ki-67 antibody was performed on xenograft tumors tissues. Brown color was positive staining (×200). (d) Expression levels of IFT88, LC3 II/I, and p62 were determined by western blot analysis of tumor tissues; the value under the band is the ratio of the blot and normalized to control.

It seems that blockage of primary ciliogenesis could enhance the malignant behaviors in HCC cells. We wanted to explore whether this phenomenon also exists *in vivo*. BALB/c nude mice were subcutaneously implanted with SMMC-7721 cells and were randomly divided into two groups until the tumor volume reached 100 mm^3^ or so. Mice were then received intratumoral injection of cholesterol-modified si-IFT88 or si-NC, respectively, at indicated time point. Tumors treated with si-IFT88 are larger than control reflected by the gross morphology after resection (Figure 5(a)). As shown in Figure 5(b), the tumor growth curves indicated that the tumors treated with si-IFT88 developed faster than control (finally, 1109.0 ± 164.0 mm^3^ vs. 668.5 ± 48.7 mm^3^; *n* = 5, *P*=0.0328). The immunohistochemical staining assay confirmed the relatively low expression of IFT88 and the relatively high level of proliferation marker Ki-67 in the tumors treated with si-IFT88 (Figure 5(c)). And the ratio of LC3 II/I was increased while the expression of p62 was decreased in si-IFT88-treated tumors compared with control groups (Figure 5(d)). These data fully indicated that blockage of primary ciliogenesis promotes tumor growth of HCC *in vivo* along with enhanced autophagic flux.

### 3.6. Overall Survival Analysis of the IFT88 and ATG7 in HCC

**Figure 6:**
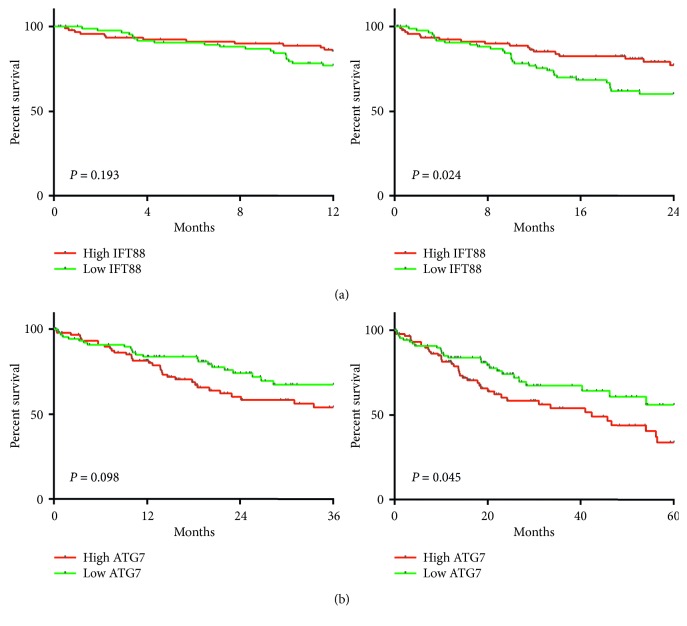
Overall survival analysis of IFT88 and ATG7 in HCC. (a) Kaplan–Meier survival analysis of IFT88 at 12 and 24 months using the Cancer Genome Atlas. (b) Kaplan–Meier survival analysis of ATG7 at 36 and 60 months using the Cancer Genome Atlas. The *x*-axis is the survival time, and the *y*-axis represents the survival rate.

To understand the clinical significance of IFT88 and ATG7 in human HCC, Kaplan–Meier survival analysis was used to learn the relationship between the expressions of IFT88 and ATG7 and the overall survival time in TCGA HCC samples. As shown in Figures 6(a) and 6(b), the patient group with IFT88 high expression or ATG7 low expression was correlated with longer survival time than the opposite group (IFT88 at 24 months, *P*=0.024; ATG7 at 60 months, *P*=0.045). Overall, these results indicated that the expression of IFT88 might be a biomarker for prognosis in HCC, while autophagy might have the protumor function in HCC.

## 4. Discussion

In 2013, two fundamental research articles published in Nature showed that ciliogenesis and autophagy come together and are intricately linked. Tang et al. found that autophagy could remove OFD1 from centriolar satellites to promote ciliogenesis in mammalian cells [34]. Pampliega et al. also proved that basal autophagy could regulate ciliary growth through the degradation of IFT88 and IFT20 in MEFs and KECs [33]. These achievements showed the context-specific roles for autophagy in the regulation of ciliogenesis and for ciliogenesis in the regulation of autophagy. Since then, several researchers have discussed the crosstalk between autophagy and primary cilia in depth. Recently, You et al. reported that IFT88 could act as a key driver of migration and invasion in liver cancer stem cells and correlates with poor prognosis in patients with HCC [44]. These important researches gave us tremendous motivation and new clues to study the interrelationship between ciliogenesis and autophagy in HCC.

In this study, the serum starvation successfully activated primary ciliogenesis in HCC cells. The length of primary cilia was longer, and the expression of IFT88 was elevated. Here, we used two HCC cell lines: SMMC-7721 cells and Huh7 cells. In both cell lines, we observed the same effects of primary ciliogenesis activated by starvation. IFT88 is an essential protein of primary ciliogenesis. As we expected, silencing IFT88 significantly decreased cilia length. Moreover, IFT88 silencing can markedly induce tumor cell proliferation, migration, and invasion. Reversely, You et al. reported that IFT88 contributed to hypoxia-induced invasion and migration in hepatoma cells [45]. IFT88 might display different function under different conditions.

Autophagy often plays a dynamic role in HCC and even promotes tumor growth and development [31, 32]. We found that blockage of primary ciliogenesis via IFT88 silencing increased the ratio of LC3 II/I, decreased p62 expression, and elevated the formation of autophagosomes and autolysosomes fluorescent puncta in HCC cells. These results strongly confirmed that IFT88 silencing suppressed the growth of primary cilia, activated autophagy, and promoted tumor properties in HCC simultaneously. It seems that the role of IFT88 in HCC cell lines was the same as it in liver cancer stem cells [44]. Recent studies suggested that PPARA and NR1H4/FXR or RPGRIP1L may play an important role in regulating cilia and autophagy in different models [46, 47]. However, the potential molecular mechanism to explain the influence of IFT88 on primary cilia and autophagy in HCC cells needs to be explored in the near future.

To unveil whether IFT88 silencing promotes tumor phenotype by activating autophagy, we used si-IFT88 and si-ATG7 to cotransfect HCC cells and then detected tumor properties. We showed that inhibition of autophagy by ATG7 silencing partially reversed the protumor effects of primary ciliogenesis blockage induced by IFT88 silencing. Furthermore, in a mouse subcutaneous tumor model, si-IFT88 treatment could obviously promote tumor growth along with the low expression of IFT88, higher level of proliferation marker Ki-67, and increasing autophagic flux. Recently, another research showed that defective ciliogenesis in thyroid Hurthle cell tumors was associated with increased autophagy [48]. It is also suggested that autophagy may contribute to tumor growth driven by defective ciliogenesis.

Finally, we used Kaplan–Meier survival analysis to understand the clinical significance of IFT88 and ATG7 in human HCC tissues and found that the high IFT88 expression group or low ATG7 expression group has significantly longer survival time than the opposite group. The primary ciliogenesis was also weakened in multiple other human cancers as mentioned above. Taken together, these results demonstrated that primary ciliogenesis obstruction promotes the malignant behaviors of HCC cells by triggering autophagy. Another inference from these results is that IFT88 may be a potential biomarker for tumor diagnosis or prognosis, which is consistent with the dysfunction of primary cilia in many human cancers.

Our results revealed that primary cilia blockage could promote the malignant behaviors of HCC and autophagy may play an important role during these processes. Given the important interrelationship of primary cilia and autophagy in cancer, further research studies need to explore the possible relationship and the possible crosstalk between them. However, Tang et al. found that starvation-induced autophagy could remove OFD1 from centriolar satellites which might represent a general mechanism to promote ciliogenesis in MCF7 breast cancer cells [34]. In ciliated cells, the presence of cilia might positively regulate autophagy, while loss of cilia is associated with upregulated autophagy in cancer cells [33, 49]. Autophagy has dynamic roles in primary ciliogenesis by degrading essential or suppressive cilia-related proteins [7]. As mentioned above, primary cilia function as signaling hubs of multiple signaling pathways relevant to cancer. However, whether and how these cilia-related signalings participate in autophagy regulation remains unclear. If autophagy plays a role in cancer through degrading cilia-related proteins, how cancer cells balance the switch to turn on/off cilia expression will be an important point to be addressed. Given the crosstalk of cilia and autophagy in cancer cells, discovery of core signaling pathway or cilia-related proteins specifically targeting these two regulators will provide more clues for cancer research.

In conclusion, we demonstrated that blockage of primary cilia induced by IFT88 silencing positively promotes the malignant behaviors of liver cancer cells by activating autophagy. IFT88 might play an important role in the intricate relationship between ciliogenesis and autophagy in HCC.

## Data Availability

The data of figures and tables used to support the findings of this study are included within the article and the supplementary information file.

## Supplementary Materials

Supplementary Materials Supplementary Table 1: sequences of siRNA. Supplementary Table 2: primer sequences used in SYBR Green qRT-PCR. Supplementary Figure: small interfering RNA (siRNA) targeting human IFT88 or ATG7 mRNA (si-IFT88 or si-ATG7) was selected.
