# Supplementary material for: Primary Cilia Blockage Promotes the Malignant Behaviors of Hepatocellular Carcinoma via Induction of Autophagy
Source: Biomed Res Int. 2019 Oct 2;2019:5202750. doi: 10.1155/2019/5202750 (PMC6791284; doi:10.1155/2019/5202750)
Supplement: Supplementary Materials — Supplementary Table 1: sequences of siRNA. Supplementary Table 2: primer sequences used in SYBR Green qRT-PCR. Supplementary Figure: small interfering RNA (siRNA) targeting human IFT88 or ATG7 mRNA (si-IFT88 or si-ATG7) was selected. [file 5202750.f1.docx]

**Supplementary materials**

### Table S1. Sequences of siRNA

| **siRNA** | **Target** **sequence** |
| --- | --- |
| **si-IFT88-1** | CGAAGCACTTAACACTTAT |
| **si-IFT88-2** | GGTGAAAGCTTCTCAATAT |
| **si-IFT88-3** | CGAAGTTCTTTACCAGATA |
| **si-ATG7-1** | GGATCCTGGACTCTCTAAA |
| **si-ATG7-2** | GAAGCTCCCAAGGACATTA |
| **si-ATG7-3** | GGAGTCACAGCTCTTCCTT |

### Table S2. Primer sequences used in SYBR Green qRT-PCR

| Gene Name |  | Sequence |
| --- | --- | --- |
| IFT88 | Forward primer | 5'-GGGTCCAAGACATCTCTGGC-3' |
|  | Reverse primer | 5'-CATGGGTCTAGTAACTCCATCCT-3' |
| ATG7 | Forward primer | 5'-CAGTTTGCCCCTTTTAGTAGTGC-3' |
|  | Reverse primer | 5'-CCAGCCGATACTCGTTCAGC-3' |
| β-actin | Forward primer | 5'-CATGTACGTTGCTATCCAGGC-3' |
|  | Reverse primer | 5'-CTCCTTAATGTCACGCACGAT-3' |

**Suppl. Figure**

**
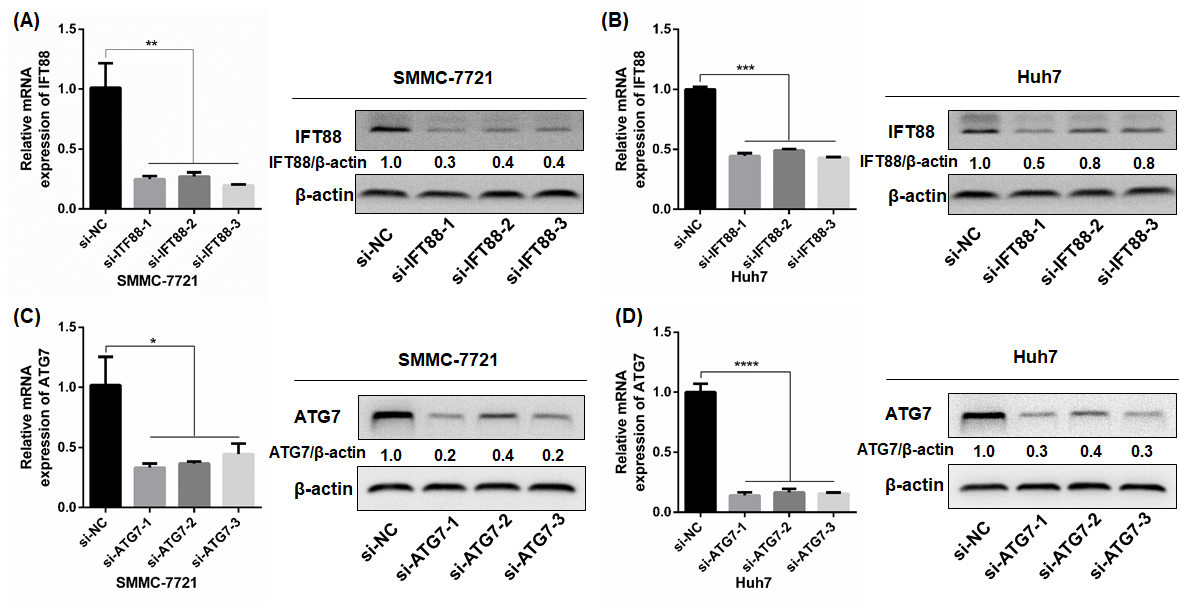
**

**Suppl. Figure 1** Small interfering RNA (siRNA) targeting human IFT88 or ATG7 mRNA (si-IFT88 or si-ATG7) was selected. A, B The RNA and protein expression of IFT88 in SMMC-7721 and Huh7 cells subjected to si-NC or si-IFT88-1, 2, 3 were determined by RT-PCR and western blot analysis; C, D The RNA and protein expression of ATG7 in SMMC-7721 and Huh7 cells subjected to si-NC or si-ATG7-1, 2, 3 were determined by RT-PCR and western blot analysis. The value under the band is the ratio of the blot and normalized to control. **P*<0.05, ***P*<0.01, ****P*<0.001, *****P*<0.0001
